# Microgravity disturbance analysis on Chinese space laboratory

**DOI:** 10.1038/s41526-019-0078-z

**Published:** 2019-07-08

**Authors:** Wenbo Dong, Wenxiang Duan, Wei Liu, Yongkang Zhang

**Affiliations:** 1 Key Laboratory 3 of Space Utilization, Chinese Academy of Sciences, 100094 Beijing, China; 2 Technology and Engineering Center for Space Utilization, Chinese Academy of Sciences, 100094 Beijing, China; 3 University of Chinese Academy of Sciences, 100094 Beijing, China

**Keywords:** Aerospace engineering, Scientific data

## Abstract

Many scientific experiments are conducted in space; therefore, it is critical to understand the microgravity environment of a space laboratory. The first Chinese cargo ship, Tianzhou-1 (TZ-1), entered space on 20 April, 2017 and later joined with the Tiangong-2 (TG-2) Chinese space laboratory. TZ-1 carried a high-precision electrostatic suspension accelerometer system (ES-ACC) for measuring the microgravity acceleration on the spacecraft and a microgravity-active vibration system (MAIS), which contained flexible quartz accelerometers (Q-ACC). The ES-ACC was able to provide a reduced-disturbance environment for the MAIS. The purpose of these two instruments was to validate novel technologies and as an opportunity to record the microgravity acceleration of TZ-1 and TG-2 in detail during spacecraft operation in different flight modes, with or without vibration isolation. The acceleration data were analyzed comprehensively in a time–frequency–amplitude spectrogram. Some periodical disturbances with orbital period and irregular signals related to certain in-orbit events were observed. After reducing those disturbances, the microgravity levels on TZ-1 and TG-2 could be resolved to better than 10^−6^ m/s^2^ in the root mean square in the frequency of 0.01–10 Hz. These accurate measurements aboard the Chinese space laboratory will provide valuable information to optimize working conditions for scientific experiments.

## Introduction

Microgravity science experiments performed on spacecraft are essential to elucidating the laws of physics (such as fluid mechanics, combustion science, materials, and fundamental physics), exploring biological phenomena, and testing novel space technology. However, disturbances on spacecraft often interfere with the microgravity environment,^1,2^ including the residual acceleration of the spacecraft orbit, the vibrations from solar panels, antennas, flywheels, fans, and astronaut activity, as well as the spacecraft attitude and orbital changes. Thus, it is important to understand the microgravity environment of the spacecraft, and if necessary, to isolate the unexpected vibrations.^3^

To understand the microgravity environment of the spacecraft, the National Aeronautics and Space Administration (NASA) set up the Principal Investigator Microgravity Services (PIMS)^4^ in order to provide scientists with acceleration analysis reports, both real time and off-line for multiple platforms, including the ISS, spaceships, satellites, sounding rockets, and microgravity towers. ZARM in Germany and NMLC in China also give the serious assessment of microgravity level for ground drop tower.^5,6^ In past space laboratory missions such as the International Microgravity Lab (IML), Mir Space Laboratory (MSL), Space Shuttle, and ISS, NASA developed the Space Acceleration Measurement System (SAMS, I and II)^7^ and the Microgravity Acceleration Measurement System (MAMS).^8^ MAMS is associated with both vibratory environment and quasi-static environment. Other similar systems include Quasi-Steady Acceleration Measurement (QSAM) developed by DLR,^9^ Microgravity Measurement Apparatus (MMA) developed by JAXA,^10^ the SIU series of accelerometers developed by Russia, and other systems. In China, supported by the China Manned Space Program, Shenzhou-4, Shenzhou-5, and Shenzhou-8 successfully carried out microgravity measurement instruments based on flexible quartz accelerometers,^11^ and later TZ-1 (the first Chinese cargo ship Tianzhou-1) carried a space electrostatic accelerometer in 2017.^12^

Measurement of both the vibration acceleration and the quasi-static microgravity environment requires accelerometers.^4,13^ For vibration acceleration (frequency from 0.01 to 300 Hz) or transient acceleration (short duration and nonperiodic), flexible quartz accelerometers (Q-ACC) are often used, such as the QA3000 from Honeywell, which has a low noise level of 10^−5^ m/s^2^ and high-bandwidth frequency. For quasi-static acceleration (frequency <0.01 Hz), electrostatic suspension accelerometers are used because their resolution can be even lower than 10^−9^ m/s^2^, in the 0.001-Hz range. The French Aerospace Lab ONERA developed the first CACTUS electrostatic suspension accelerometer, which had a resolution of 10^−9^ m/s^2^, early in the 1970s, and then many accelerometers based on this technology, such as ASTRE,^14^ STAR, SuperSTAR, GRADIO, and MESA^8^ were developed and employed for different satellites. The highest resolution of such an accelerometer is 10^−12^ m/s^2^, and its main uses involve fundamental physics, such as the measurement of earth’s gravity field and tests of the equivalence principle. In China, Huazhong University of Science and Technology (HUST) developed space electrostatic accelerometer HSEA-1,^15,16^ which was first tested in a scientific satellite in 2006, and later successfully used in TZ-1 in 2017. The microgravity data on SJ-10 in 2016 show a better microgravity environment, which has a level of 10^−5^ m/s^2^.^17^

An issue with electrostatic accelerometers can be their high sensitivity (normally <10^−3^ m/s^2^), so a small disturbance to the spacecraft may drive it into saturation. To isolate the disturbance and provide a reduced-disturbance platform, MAIS, which is six-degree-of-freedom electromagnetic technology, has been adopted to attenuate the vibration. It has the vibration isolation ability of 0–40 dB in the frequency of 0.03–200 Hz, and its active control ability could also provide calibration excitation signals for ES-ACC. Chinese Academy of Sciences (CAS) started related research since 2010^18–20^ and MAIS was successfully applied on TZ-1. MAIS also has two three-axis Q-ACCs for measuring vibrations across larger measuring ranges to the level better than 7.5 × 10^−1^ m/s^2^ and wider frequency bands of 0–250 Hz.

In this report, the measurement data recorded by ES-ACC and Q-ACC are analyzed. First, the real microgravity level, which is on-orbit measured on TG-2 and TZ-1 in different flight modes, is given for assessing the real microgravity condition on a Chinese space laboratory. Second, the regular or irregular disturbance signals are studied and some are recognized to be relative with on-orbit events. Finally, the cooperation working principle and installation of ES-ACC and MAIS is also presented.

## Results

### Acceleration data formation

For microgravity measurement and analysis, the Microgravity Environment Interpretation Tutorial (MEIT)^21,22^ provides important references^2,22^ and examples of data analysis. Our methods of analyzing microgravity acceleration data work in the time domain and frequency domains. The time-domain data include acceleration by ES-ACC with 20 points per second, and acceleration by Q-ACC with 250 points per second, as well as relative position/angle data and magnetic force/torque data. In the frequency domain, the single sequence is converted by a discrete Fourier transform into an amplitude analysis spectrum (AFS) and power- spectrum density (PSD), where AFS is suitable for the deterministic signal, and PSD is more suitable for random noise signals. In the microgravity specification SSP41000,^23^ the frequency data are summarized to the root mean square (RMS) in one-third octave band (OTOB), or even the 1/96 octave band,^4,21^ for expressing the level of both the deterministic signal and random noise.

**Fig. 1:**
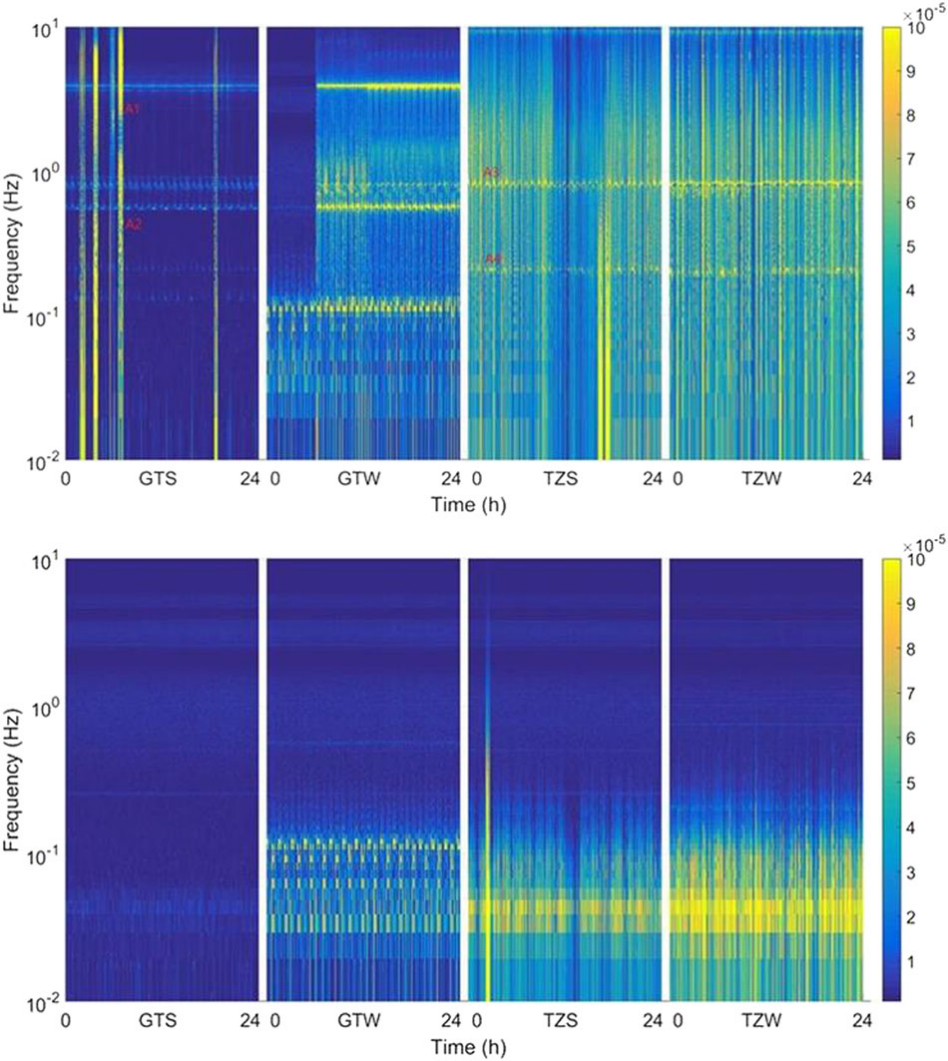
**a** Microgravity spectrogram measured by ES-ACC in different flight modes (colorbar: ${\mathrm{m}}/{\mathrm{s}}^2/\sqrt{\mathrm{Hz}}$): (1) GTS (30-04-2017), (2) GTW (16-05-2017), (3) TZS (24-06-2017), and (4) TZW (30-08-2017). **b** Microgravity spectrogram measured by ES-ACC with vibration isolation (colorbar: ${\mathrm{m}}/{\mathrm{s}}^2/\sqrt{\mathrm{Hz}}$): (1) GTS (09-05-2017), (2) GTW (13-05-2017), (3) TZS (28-06-2017), and (4) TZW (03-09-2017)

**Fig. 2:**
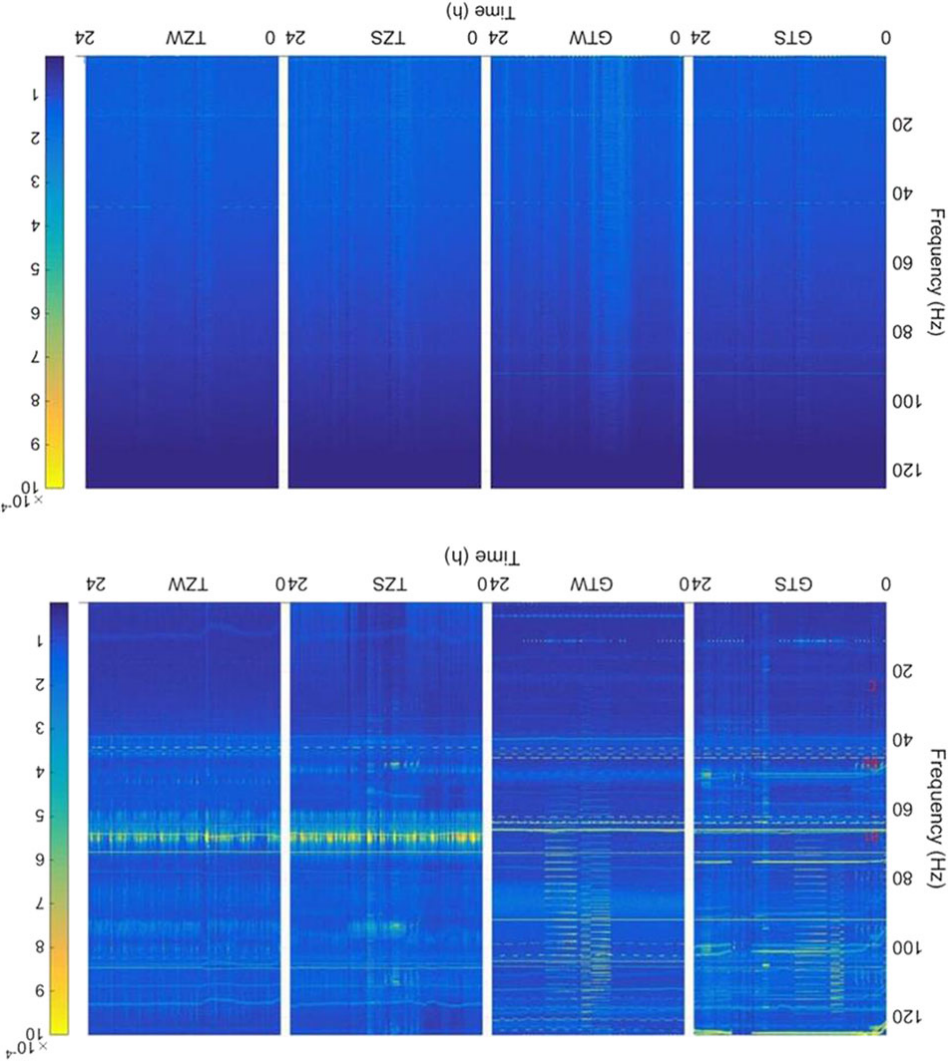
**a** Disturbance spectrogram measured by Q-ACC in different flight modes (colorbar: ${\mathrm{m}}/{\mathrm{s}}^2/\sqrt{\mathrm{Hz}}$): (1) GTS (30-04-2017), (2) GTW (16-05-2017), (3) TZS (24-06-2017), and (4) TZW (30-08-2017). **b** Disturbance spectrogram measured by Q-ACC after vibration isolation (colorbar: ${\mathrm{m}}/{\mathrm{s}}^2/\sqrt{\mathrm{Hz}}$): (1) GTS (09-05-2017), (2) GTW (13-05-2017) (3) TZS (28-06-2017), and (4) TZW (03-09-2017)

Combining the time domain and frequency domain, the time “Cfrequency” Camplitude Spectrogram^22^ is more intuitive for long-period analysis, which uses time as the X-axis and frequency as the Y-axis, and color as the amplitude of AFS or PSD, where we can see the whole frequency response in one figure and find more information. In this paper, the disturbance signals are mainly studied based on the spectrogram, as shown in Figs. 1 and 2.

### Acceleration measurement results by ES-ACC and Q-ACC

TZ-1 was launched on 20 April, 2017 and operated until 5 September, 2017, which is about 139 days. During that time, MAIS and ES-ACC were operated for 84 days in four working modes, defined as GTS, GTY, TZS, and TZY. GTS means TG-2 and TZ-1 assembly working in a stabilized orientation mode, from 04.29 to 05.12 and 06.01 to 06.03; GTY means TG-2 and TZ-1 assembly working in yaw-steering mode, from 05.12 to 05.23 and 05.29 to 06.01; GTS means TZ-1 independent working in a stabilized orientation mode, from 06.23 to 07.01 and 07.22 to 08.26; and GTY means TZ-1 independent working in yaw-steering mode, from 08.26 to 09.04.

### Microgravity measurement by ES-ACC

Figure 1 shows a typical spectrogram of power spectrum density (PSD) curves for 24 h, respectively, in GTS, GTW, TZS, and TZW. Figure 1a represents the original disturbance on the spacecraft, while Fig. 1b is the microgravity level after the isolation of MAIS. From these curves, it can be found that

- The microgravity level on the TG-2 and TZ-1 assembly (GTS and GTW) is better than TZ-1 (TZS and TZW), because TG-2 uses low-noise CMGs as actuators, and TZ-1 independent uses a gas-pulse thruster as an actuator. The thruster force produces more noise and transient disturbance, so the microgravity becomes worse. In Fig. 1a (3) and (4), there are some vertical bright lines, which are caused by the thruster disturbance that exceeds the range of ES-ACC. Only after vibration isolation, can such disturbance be reduced.
- The microgravity level in the stabilized orientation mode (GTS) is better than in the yaw-steering mode (GTW), because the yaw steering of the spacecraft will cause larger vibrations and magnify the natural frequency (0.8 and 4 Hz).
- MAIS can isolate spacecraft vibrations and provide an isolation environment. In Fig. 1b, the spectrogram after vibration isolation is much smoother and the microgravity environment is improved with the help of MAIS. However, it only attenuates the high-band frequency noise and does not affect the low-band acceleration measurement of ES-ACC.

### Disturbance measurement by Q-ACC

Besides ES-ACC, MAIS also has two three-axis Q-ACCs to measure acceleration, one is on the stator and the other on the floater. The precision of Q-ACC (more than 10^−5^ m/s^2^) is worse than ES-ACC, but the measuring range (−0.75–0.75 m/s^2^) and frequency band (0–125 Hz) is much larger, which is an important supplement for measurement of ES-ACC.

Figure 2 shows the acceleration spectrogram with the same duration as that shown in Fig. 1. The frequency axis is set for a linear scale, so that the resolution is easier to see. Though the precision is not better than that of ES-ACC, it contains more information about on-orbit disturbances in the high-band frequency, like the bright lines in Fig. 2a.

## Discussion

**Fig. 3:**
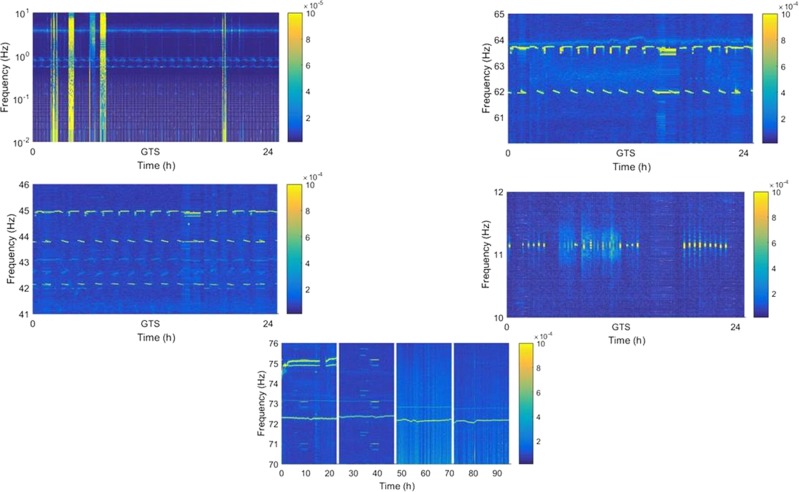
Magnified detailed feature of disturbance signals. **a**–**d** are the multiple PSD traces (colorbar: ${\mathrm{m}}/{\mathrm{s}}^2/\sqrt{\mathrm{Hz}}$): **a** the feature of solar panels, **b**, **c** the feature of antenna actuation, **d** the feature of a special payload, and **e** the feature of a cooling fan and an air conditioner

From the spectrogram in Figs. 1a and 2b, many regular and irregular signals can be found and recognized as the effects of spacecraft actuation. For more detailed analysis, some signals are MAGNIFIED in Fig. 3.

As shown in Fig. 1a, we have seen some bright horizontal lines at 4 Hz in the GTS and GTW mode, which is the inherent mechanical frequency point of TG-2 and TZ-1 assembly according to spacecraft reports.

Magnifying the spectrogram in Fig. 1a, more details can be found. As shown in Fig. 3a, we can see some zigzag bright lines at 0.6 and 0.1 Hz in the GTS and GTW mode and 0.8 and 0.1 Hz in the TZS and TZW mode. The frequency point line is not straight, but gradually fluctuates with a period of 92 min, that is, the spacecraft orbit period. It is inferred to be a feature of solar panels, which are always driven to point to the sun gradually. Considering that the length of the solar panel TG-2 is 18.4 m and that of TZ-1 is 14.9 m, it is reasonable that the line of 0.6 Hz appeared when TZ-1 joined with TG-2, while 0.8 Hz is for TZ-1.

As shown in Fig. 3b, c, there are also some periodic signals at 62–64 and 42–45 Hz, with periods of about 92 min, the period of the spacecraft orbit. These signals are not continuous but rather segmented as discrete lines. From the spacecraft operation diary, this feature is consistent with antenna actuation. When the spacecraft enters the monitoring area, the communication antennas need to be regulated to point at the correct angle and then return to the original angle when exiting. For this reason, the frequency point changes every 92 min.

Compared with the data of different modes, 62–64 Hz should be the feature of antennas of TG-2 and 42–45 Hz should be the feature of antennas of TZ-1.

There is also some periodic actuation in the frequency points of about 11.2 Hz, as shown in Fig. 3d. They did not appear throughout the entire 24 h, but only at the intervals corresponding to working time, such as 9:00 a.m. to 12:00 a.m.

It may be caused by actuation of this special payload that requires more communication or interaction with the ground, such as telemetry or tele-control operations, though which payload is not clear.

From engineering data, the temperature of TZ-1 changes by more than several degrees celsius. In order to control the thermal environment, there are many cooling fans or air conditioners on TG-2 and TZ-1. Figure 3e shows the difference of the system noise when two cooling fans are open (in GTS mode) and closed (in GTW mode). From the spectrum in the figure, there are obvious frequency lines at around 75.1 and 74.9 Hz, which is affirmatively the noise point of cooling fans according to the ground test.

Another frequency line is at about 72 Hz, which is certainly affected by an air conditioner. Since the temperature is always alternating in the spacecraft, the bright line is not straight but changes according to temperature change. This is also in accordance with the test on the ground.

Some of the above analysis results have been checked with the spacecraft diary, and some analysis only is by reasonable inference. Still, this study is necessary and heuristic because only during in-orbit working does it show the real conditions of the spacecraft, the accurate frequency, and amplitude of disturbances and changes in the orbit. These cannot be obtained during the the ground test without interference.

As a summary, we can present the disturbance feature on the spacecraft as Table 1.

**Table 1:** Disturbance feature on TG-2 and TZ-1

Events (×10^−5^ m/s^2^/Hz^1/2^)		Frequency point (Hz)	Amplitude (*X*)	Amplitude (*Y*)	Amplitude (*Z*)	Amplitude
A Mechanical vibration of the spacecraft and solar panel	(TG-2)	4 Hz	0.5899	0.6895	0.3844	0.9721
	(TG-2)	0.6 Hz	2.531	0.8585	4.199	4.540
	(TZ-1)	0.8 Hz	4.817	4.381	5.259	7.953
B Antenna actuation	(TG-2)	6264 Hz	12.53	19.01	12.6	23.76
	(TZ-1)	4245 Hz	6.199	7.562	9.563	15.55
C Special payload disturbance	(TG-2)	11.2 Hz	12.12	21.04	5.831	25.24
	(TZ-1)	0.9 Hz	5.778	5.312	4.209	8.514
D Cooling fan vibration	(TG-2)	75 Hz	75.35	11.03	122.7	136
	(TZ-1)	72 Hz	10.02	5.791	22.14	24.95

**Fig. 4:**
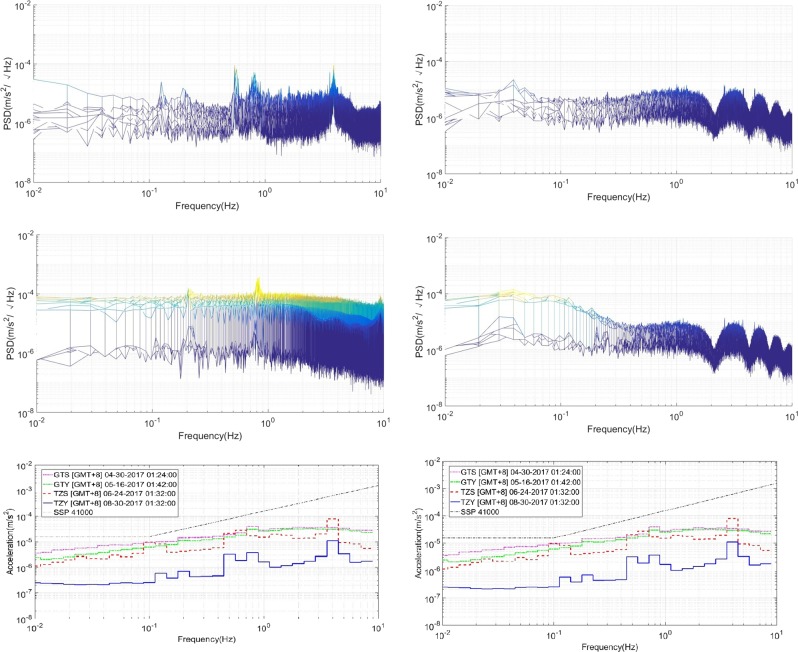
Microgravity acceleration assessment on TG-2 and TZ-1 in PSD. **a** Original microgravity level in the GTS mode. **b** Microgravity level after vibration control in the GTS mode. **c** Original microgravity level in the TZS mode. **d** Microgravity level after vibration control in the TZS mode. Microgravity acceleration assessment on TG-2 and TZ-1 in RMS of OTOB. **e** Original microgravity level on different modes (RMS). **f** Microgravity level after vibration control on different modes (RMS)

It is necessary to assess the microgravity level on TG-2 and TZ-1. The PSD spectrum in the working mode of GTS and TZS is drawn as Fig. 4a–d. If, according to the specification of SSP4100D, the PSD spectrum should be present as the RMS spectrum in one-third octave band (OTOB), as shown in Fig. 4e, f. ES-ACC measured the microgravity at least below 10 Hz.

The original microgravity on TG-2 and TZ-1 assembly is about 10^−5^ to 10^−4^ m/s^2^ (RMS). After vibration isolation, it could be improved to, at best, 0–40 dB at the frequency from 0.03 Hz, and the microgravity will be on the order of 10^−6^ to 10^−7^ m/s^2^ at the quietest state. Comparison to ISS, MSL, and other experimental satellites shows TZ-1 and TG-2 assembly to be one of the best platforms for microgravity experiments.

It should be noted that this assessment does not include the quasi-static acceleration of 0 Hz, which is determined by spacecraft rotation, atmosphere drag force, solar radiation light pressure, and similar factors, normally below 10^−6^ m/s^2^ in the space laboratory.

As a conclusion, microgravity measurement and disturbance analysis on the Tianzhou-1 Cargo Ship was introduced with the instrument of ES-ACC and MAIS. The microgravity disturbance is measured by the electrostatic suspension accelerometer and a flexible quartz accelerometer, and the data are analyzed comprehensively with the whole time–frequency–amplitude spectrogram. By analyzing the signals with periodical features, the spacecraft disturbances and some irregular events are recognized, including the mechanical vibration, solar panel movement, antenna actuation, special payload actions, cooling fan switching, and so on. The accurate amplitude value and frequency points of all these disturbances are summarized, which is important for understanding the real vibrations of TG-2 and TZ-1 in-orbit. Moreover, MAIS successfully isolated the disturbance for ES-ACC and the latter gave a quantified assessment of the microgravity level, which is at a level of 10^−6^ m/s^2^ in RMS of OTOB.

## Method

### System design and installation

**Fig. 5:**
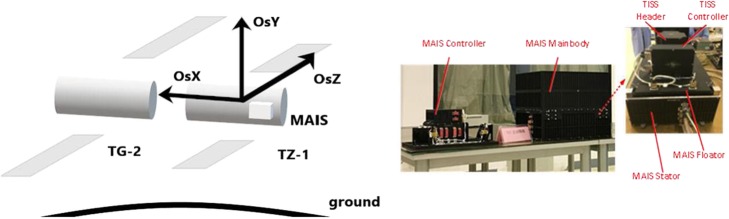
MAIS and ES-ADD installation on TZ-1

MAIS and ES-ACC are combined together, as shown in Fig. 5. The in-orbit experimental instrument has two parts: the MAIS controller and the MAIS main body (composed of the MAIS stator and MAIS floater). The payload ES-ACC, which is composed of the ES-ACC controller and ES-ACC header, is installed on the MAIS floater inside the main body, and all of them are protected in a shielding case. The floater is locked on the stator and could be unlocked after entering the orbit. The stator and floater are isolated by electromagnetic suspension, so that the floater and ES-ACC are less affected by the vibrations on the spacecraft.

**Fig. 6:**
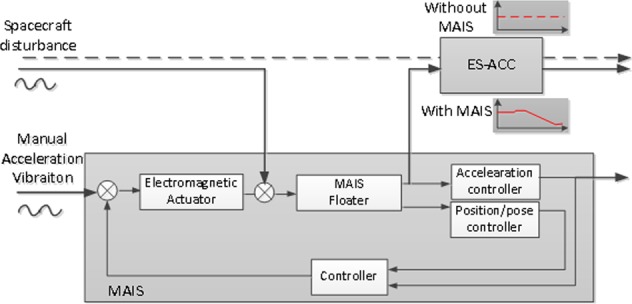
Cooperative working principle of MAIS and ES-ACC

The system is installed on TZ-1 as shown in Fig. 6. The MAIS main body is fixed on the fourth quadrant in the rear, near the centroid of the spacecraft, so that its vibration environment is less affected by spacecraft attitude control. The centroid changes after TZ-1 joins with TG-2. The coordinate system is defined as follows: *X* is the launching direction, and *Y* and *Z* are, respectively, the horizontal and vertical directions on TZ-1.

### Working principle of cooperation

ES-ACC consists of a sensor head, a displacement transducer, a controller, and an actuator. The sensor head consists of a proof mass (PM) and its surrounding electrode cage, where the six-DOF motions of the PM are measured by a six-channel capacitive displacement transducer. The low-frequency feedback voltages calculated from the controller are applied on the electrodes by drive-voltage amplifiers. Finally, the PM is held motionless with respect to the cage. In this case, the feedback voltage can indicate the differential forces acting on the PM, as well nongravitational forces or accelerations acting on the spacecraft.^15^ The intrinsic noise of ES-ACC is tested at about 10^−9^ m/s^2^ in the ground.

MAIS works by the principle of active electromagnetic suspension. The controller sample acceleration signals from its Q-ACC and relative position signals from the position sensor detector, calculates the feedback current, and then drives electromagnetic coils to compensate the disturbance.^19,20^ The close-loop control algorithm includes two kinds of strategies: position-following control uses the relative position as feedback, while acceleration-following control directly uses acceleration signals as feedback, but the relative position only to avoid collisions. The effect of active control is actually like a low-stiffness magnetic spring with the low-band frequency point. Some similar active vibration isolation systems have been developed within recent years,^24^ including STABLE,^25^ g-LIMIT,^26^ MIM and MIM-2,^27^ MVIS,^28^ and ARIS,^29^ that have been tested on MSL, Space Shuttle, or ISS. Other space optical systems,^30,31^ such as SUITE,^32^ VISS,^33^ and DFP,^34^ also used similar technology for low-frequency mechanical vibration control.

When the two systems work together, MAIS is equivalent to a structure filter as shown in Fig. 6, which attenuates the high-frequency noise for ES-ACC, without changing the magnitude of the low-frequency disturbance. The theoretical cutoff frequency of MAIS can be configured according to the requirements, and its isolation ability is 0–40 dB at a frequency from 0.03 to 125 Hz. For ES-ACC, the absolute measuring range is 0–10 Hz, and its range is about 2 × 10^−5^ m/s^2^. If the disturbance exceeds the measuring range of ES-ACC, MAIS starts to work to ensure that it is working normally. Though MAIS also brings some structure noise, it does not affect the low-band measurement result of ES-ACC.

MAIS also imposes the manual vibration input at the floater, together with the payload. Suppose the given position input is *x* = *A*sin(2*πft*), then the acceleration curve on ES-ACC is *a* = (4*π*^2^*f*^2^)*A*sin(2*πft*). For example, this function could be used for frequency sweep and calibration of ES-ACC or provided the required specific vibration for other scientific experiments.

### Reporting summary

Further information on research design is available in the Nature Research Reporting Summary linked to this article.

## Supplementary information

Reporting Summary Checklist

## Data Availability

All the relevant data and codes are available from the authors.

## Supplementary information

**Supplementary Information** accompanies the paper on the *npj Microgravity* website (10.1038/s41526-019-0078-z).

## Code availability

All codes are available from the authors upon reasonable request.
